# Transcriptome analysis of *Bipolaris sorokiniana* - *Hordeum vulgare* provides insights into mechanisms of host-pathogen interaction

**DOI:** 10.3389/fmicb.2024.1360571

**Published:** 2024-03-20

**Authors:** Poulami Basak, Malkhan Singh Gurjar, Tej Pratap Jitendra Kumar, Natasha Kashyap, Dinesh Singh, Shailendra Kumar Jha, Mahender Singh Saharan

**Affiliations:** ^1^Division of Plant Pathology, ICAR-Indian Agricultural Research Institute, New Delhi, India; ^2^Division of Genetics, ICAR-Indian Agricultural Research Institute, New Delhi, India

**Keywords:** *Hordeum vulgare* (barley), transcriptome, differentially expressed genes, resistant gene analogs, metabolism, effector

## Abstract

Spot blotch disease incited by *Bipolaris sorokiniana* severely affects the cultivation of barley. The resistance to *B*. *sorokiniana* is quantitative in nature and its interaction with the host is highly complex which necessitates in-depth molecular analysis. Thus, the study aimed to conduct the transcriptome analysis to decipher the mechanisms and pathways involved in interactions between barley and *B*. *sorokiniana* in both the resistant (EC0328964) and susceptible (EC0578292) genotypes using the RNA Seq approach. In the resistant genotype, 6,283 genes of *Hordeum vulgare* were differentially expressed out of which 5,567 genes were upregulated and 716 genes were downregulated. 1,158 genes of *Hordeum vulgare* were differentially expressed in the susceptible genotype, out of which 654 genes were upregulated and 504 genes were downregulated. Several defense-related genes like resistant gene analogs (RGAs), disease resistance protein RPM1, pathogenesis-related protein PRB1-2-like, pathogenesis-related protein 1, thaumatin-like protein PWIR2 and defensin Tm-AMP-D1.2 were highly expressed exclusively in resistant genotype only. The pathways involved in the metabolism and biosynthesis of secondary metabolites were the most prominently represented pathways in both the resistant and susceptible genotypes. However, pathways involved in MAPK signaling, plant-pathogen interaction, and plant hormone signal transduction were highly enriched in resistant genotype. Further, a higher number of pathogenicity genes of *B*. *sorokiniana* was found in response to the susceptible genotype. The pathways encoding for metabolism, biosynthesis of secondary metabolites, ABC transporters, and ubiquitin-mediated proteolysis were highly expressed in susceptible genotype in response to the pathogen. 14 and 11 genes of *B*. *sorokiniana* were identified as candidate effectors from susceptible and resistant host backgrounds, respectively. This investigation will offer valuable insights in unraveling the complex mechanisms involved in barley- *B. sorokiniana* interaction.

## 1 Introduction

Spot blotch incited by *Bipolaris sorokiniana* (Teleomorph: *Cochliobolus sativus*) is a significant disease affecting barley production. Barley in grown worldwide in an area of 47.14 million hectares with an annual production of 154.88 million tonnes (FAO, 2022). In India, barley occupies an area of 0.45 million hectares with an annual production of 1.37 million tonnes (FAO, 2022). The disease is considered a serious threat in the hot and humid South-Asian regions (Chaurasia et al., 2000; Kumar et al., 2007; Chand et al., 2008; Vaish et al., 2011). A yield loss of 25–45% has been reported in barley due to the pathogen, which has the potential to escalate even more under conducive environments (Iftikhar et al., 2009). In Indian context, yield losses can extent to 53% (Vaish et al., 2011). Changes in agricultural methods and the quick substitution of locally adapted varieties with high-yielding cultivars have resulted in the emergence of spot blotch (Bala and Kaur, 2008). This disease poses a threat to barley cultivation in the future and can cause devastating losses under the climate change scenario.

*Bipolaris sorokiniana* is a hemi-biotrophic pathogen causing spot blotch on foliar parts, black point on grains, and root rot symptoms. The symptoms are characterized by necrotic lesions accompanied by chlorosis, severe cases lead to the complete drying of leaves. The pathogen is seed, soil-borne and can spread by air-borne conidia making it one of the most destructive pathogens (Gupta et al., 2018). It produces thick-walled conidia and has a wide host range attacking various other cereals and weed hosts. This pathogen shows high genetic variability which can be attributed to parasexuality and heterokaryosis (Glass et al., 2000). The pathogenicity determinants of *B*. *sorokiniana* include factors like higher melanin content, cell wall degrading enzymes, and the presence of toxic compounds (Jahani et al., 2006; Chand et al., 2014; Aich et al., 2017). The genome size of *Bipolaris sorokiniana* (ND90Pr) is 34.42 Mb (Condon et al., 2013). Being a hemibiotrophic pathogen, it consists of an early biotrophic phase where effector proteins are employed to counteract host defense responses (Kaladhar et al., 2023). In the wheat pathosystem, effectors like *BsToxA*, *CsSp1and BsCE66* have been found to govern virulence in *B*. *sorokiniana* (Friesen et al., 2018; Zhang et al., 2022; Kaladhar et al., 2023). Few studies have also reported the *VHv1* gene to be responsible for governing virulence in barley (Zhong et al., 2002).

This disease can be managed by the use of chemical fungicides belonging to the triazole group. However, as chemicals cause a negative impact on the environment, the use of resistant cultivars remains the most feasible option. Few genes like *Rcs 5*, *Rcs* 6, and a few QTLs have been identified in governing resistance to spot blotch in barley. Some reports suggested that *mlo* gene-based resistance to powdery mildew caused by *Blumeria graminis* results in susceptibility to *B*. *sorokiniana* in barley (Kumar et al., 2001; Leng et al., 2020). However, the presence of complex factors and the quantitative nature of spot blotch resistance makes breeding programs challenging and time-consuming. Additionally, the classical gene for gene model is not the principal system operating in the *H. vulgare*-*B*. *sorokiniana* pathosystem (Ghazvini and Tekauz, 2008). Thus, there is a need for in-depth elucidation of genetic factors responsible for governing resistance. In addition, the elucidation of pathogen factors responsible for virulence will provide greater insights into the molecular basis of *B*. *sorokiniana*-*Hordeum vulgare* interaction.

Keeping in view, we investigated the transcriptome of resistant and susceptible genotypes of barley with challenge inoculation of *B*. *sorokiniana* to investigate the mechanisms associated and identified a few defense-related genes. In addition, differentially expressed genes (DEGs), pathways involved in defense response, and pathogenicity factors involved in virulence were identified. The present investigation will offer valuable understanding in unraveling the complex pathogenic mechanisms involved in barley- *B*. *sorokiniana* interaction.

## 2 Materials and methods

### 2.1 Fungal material

A virulent isolate (BS 52, NCBI accession number OR262940) of *B*. *sorokiniana* inciting spot blotch of barley was established in the Fungal Molecular Biology Laboratory, Division of Plant Pathology, ICAR-Indian Agricultural Research Institute, New Delhi. *B*. *sorokiniana* was maintained in test tubes containing Potato dextrose agar media in an incubator at 25°C. Inoculum was multiplied by inoculation of *B*. *sorokiniana* in sorghum grains for 15–20 days at 25°C followed by preparation of spore suspension (10^4^ conidia/ml). The *in-vitro* transcriptome of *B*. *sorokiniana* was assessed and compared to the *in-planta* transcriptome. For pathogen mycelia, *B*. *sorokiniana* was inoculated in potato dextrose broth media and kept at 25°C in a shaker incubator (Kuhner Lab-Therm). The fungal mycelia were extracted after 10 days of incubation and kept in a deep freezer (−80°C) until further study.

### 2.2 Experimental genotypes and growth conditions

The resistant (EC0328964) and susceptible (EC0578292) genotypes (ICAR-NBPGR, New Delhi) were taken for RNA-seq analysis. The genotypes were sown in 4-inch pots containing an equal proportion of sand, soil and FYM under net house conditions. Two replications for each treatment (control and inoculated) were maintained. After 30 days, the plants were inoculated with *B. sorokiniana* (BS 52) spore solution (10^4^ conidia/ml) and kept in a moisture chamber (100% humidity). The control plants were inoculated with sterile distilled water. After inoculation, leaves were sampled at 0 h, 12 h, 24 h, and 36 h and kept in aluminum foil, and immediately frozen in liquid nitrogen. Then the inoculated leaves were kept in a deep freezer (−80°C) for further study.

### 2.3 RNA isolation, library preparation, and RNA sequencing

The transcriptome analysis was performed for resistant and susceptible genotypes of barley with challenge inoculation of the pathogen. Two biological replicates for each treatment were considered for the RNA sequencing. Total RNA was extracted from the leaves and the *B*. *sorokiniana* mycelia using the Trizol method with slight modifications. The double-strand cDNA quality was checked using the Qubit dsDNA estimation and agarose gel. Pooling of the samples (different time points) for each treatment was done. The libraries for all the samples were prepared using the NEB Next UltraII DNA library preparation kit for the Illumina sequencing. The DNA underwent fragmentation to achieve an insert size of approximately 300–400 base pairs per sample. Subsequently, the fragmented DNA was repaired at the ends, followed by A-tailing, and ligation of indexed adapters. The resulting products were purified, and PCR amplification was conducted to produce the final library. The quantification of the library was carried out using the Qubit Fluorometer (Invitrogen, Life Technologies, Grand Island, NY, USA). The distribution of fragments within the library was checked on the HSDNA kit using the Tape station (Agilent Technologies, USA). The DNA libraries, now tagged, were combined in equal proportions, and loaded onto the c-bot automated system for the cluster generation. The sequencing was conducted in the S4 flow cell of the Illumina NOVASEQ 6000 platform by 150 bp paired-end. Post-sequencing, the samples were demultiplexed, and the sequences associated with indexed adapters were removed using the CASAVA v1.8.2 software (Illumina Inc.).

### 2.4 Read quality check and adapter trimming

The FastQC software was used to perform a quality assessment of the reads. The parameters like base quality score distribution, sequence quality score distribution, average base content per read and GC distribution in the reads were examined. The Universal Illumina Adapters (AGATCGGAAGAGC) were eliminated using the trim galore (version 0.6.2), and sequences shorter than 20 base pairs were excluded. Apart from removing adapters, low-quality ends of the reads were trimmed, maintaining a phred score of 20.

### 2.5 Differential gene expression, functional annotation, and effector prediction

The reference genomes of *Hordeum vulgare* (Morex; *GenBank* assembly: GCA_904849725; Mascher et al., 2021) and *B*. *sorokiniana* (ND90Pr; *GenBank* accession: AEIN00000000; Condon et al., 2013) were used. Indexing of these reference genomes were conducted through BWA (version 0.7.5). The adapter-trimmed sequences were aligned to the *Hordeum vulgare* reference genome using the bwa mem algorithm with default parameters (version 0.7.5). The mapped and unmapped sequences were separated using the SAMTOOLS (version 0.1.19). The unmapped sequences were subsequently aligned to the reference genome of *B*. *sorokiniana* using the SAMTOOLS. The count of reads mapped to genes was determined using the SAMTOOLS (version 0.1.19). For differential gene expression analysis, all possible combinations were assessed using the DESeq (version 1), an R package. Transcripts exhibiting a minimum two-fold difference in the gene expression and a *p*-value of ≤0.05 were selected for further analysis. The gene annotation was performed using the Blast2GO^1^ (Gotz et al., 2008) and the pathways were identified using the KEGG database^2^ (Kanehisa and Goto, 2000). For the prediction of effectors from the *B*. *sorokiniana* transcripts, the following prediction pipeline is used sequentially (Zhang et al., 2020). The transcripts were checked for the presence of N-terminal signal peptide using the Signal P5.0 server.^3^ The selected sequences were then checked for subcellular localization using the Target P2.0 server^4^ followed by TMHMM 2.0 server^5^ to check for the presence of transmembrane domain. The filtered sequences were checked with the EffectorP 3.0 server^6^ for the prediction of effectors.

## 3 Results

### 3.1 Data statistics and mapping of reads generated from RNA-sequencing

To understand the mechanisms and key genes involved during *B*. *sorokiniana* infection in barley, transcriptome analysis was performed using the RNA Seq approach. A total of 333.40 million reads were generated from Illumina sequencing. The raw reads were deposited to the NCBI SRA database (Accession no: PRJNA996376). The reads were mapped to the reference genome of barley (Morex) and the unmapped reads were then mapped to the reference genome of *B*. *sorokiniana* (ND90Pr). The reads ranged from 13.6 million to 77.7 million across samples. The mapping percentage ranged from 72.79 to 98.55% having a read length of 159 bp. The GC content ranged from 48–54% (Table 1).

**Table 1:** Statistics of sequenced and mapping data of *Hordeum vulgare* and *B. sorokiniana*.

Particulars	No. of raw reads	Number of bases	Mapped reads	Unmapped reads	% GC content	Raw read length (bp)
RI1	32878316	10455304488	93.58%	6.42%	52	159
RI2	20835211	6625597098	94.23%	5.77%	51	159
RC1	25703788	8173804584	98.44%	1.56%	50	159
RC2	13670079	4347085122	98.55%	1.45%	50	159
SI1	18665786	5935719948	93.83%	6.17%	48	159
SI2	39226542	12474040356	93.37%	6.63%	51	159
SC1	35229653	11203029654	96.65%	3.35%	54	159
SC2	77761704	24728221872	97.75%	2.25%	54	159
BS 1	36177912	11504576016	76.44%	23.56%	49	159
BS 2	33257117	10575763206	72.79%	27.21%	50	159

RI, Resistant Inoculated; RC, Resistant Control; SI, Susceptible Inoculated; SC, Susceptible Control; BS, Bipolaris sorokiniana grown in-vitro.

### 3.2 Analysis of genes of *Hordeum vulgare*

#### 3.2.1 Differential gene expression analysis (DEGs)

**Figure 1:**
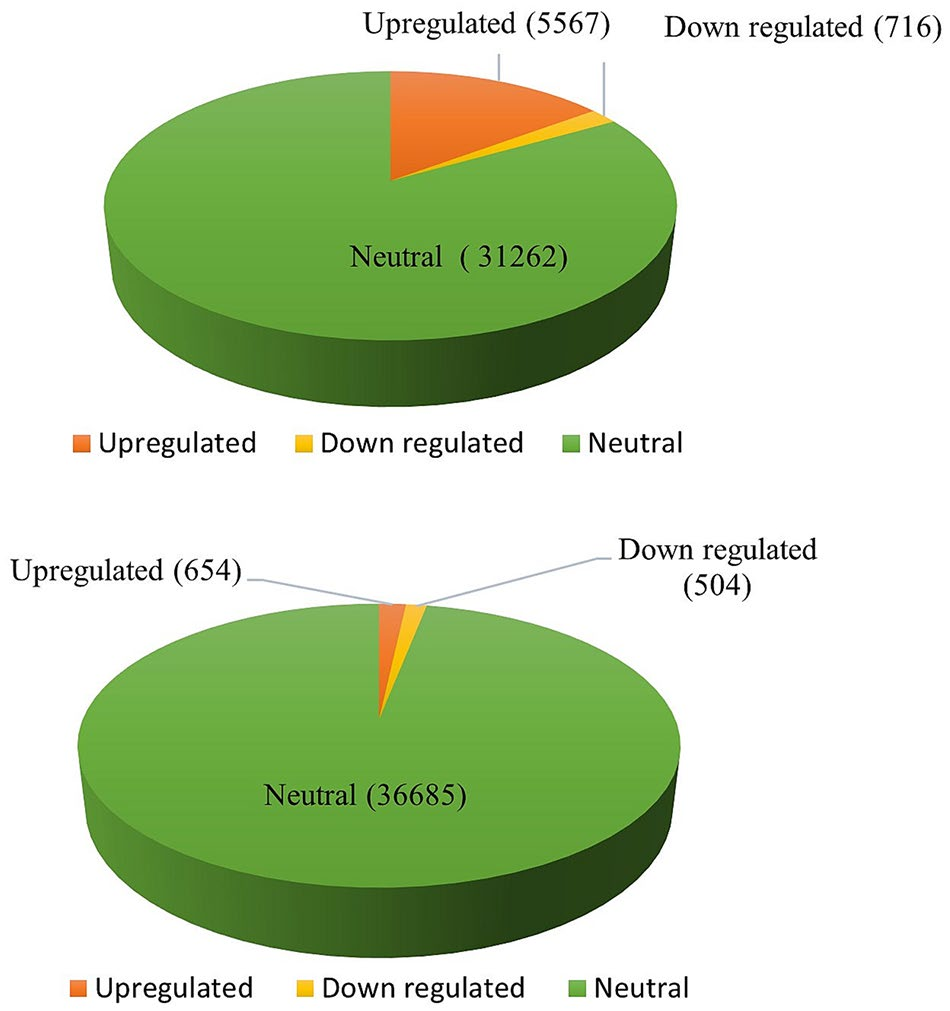
Differential gene expression analysis (top) resistant genotype (EC0328964) and (bottom) susceptible genotype (EC0578292) of barley upon infection of *B. sorokiniana*.

**Figure 2:**
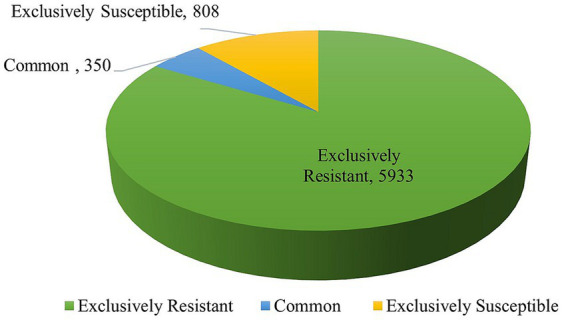
Common and unique genes between resistant and susceptible genotypes of barley upon infection of *B. sorokiniana*.

DEGs were assessed in both the combinations of resistant control-resistant inoculated (RC_RI) and susceptible control-susceptible inoculated (SC_SI). In the resistant genotype (EC0328964), 6,283 genes were differentially expressed with a *p*-value of ≤0.05 and log2 fold, out of which 5,567 genes were upregulated and 716 genes were downregulated. In the susceptible genotype (EC0578292), 1,158 genes were differentially expressed with a *p*-value of≤0.05 and log2 fold, out of which 654 genes were upregulated, and 504 genes were downregulated (Figure 1). Between resistant and susceptible combinations, 350 genes were commonly expressed, 5,933 genes were exclusively expressed in resistant genotype and 808 genes were exclusively expressed in the susceptible genotype (Figure 2) (Supplementary Table S1).

#### 3.2.2 Gene ontology and functional annotation

**Figure 3:**
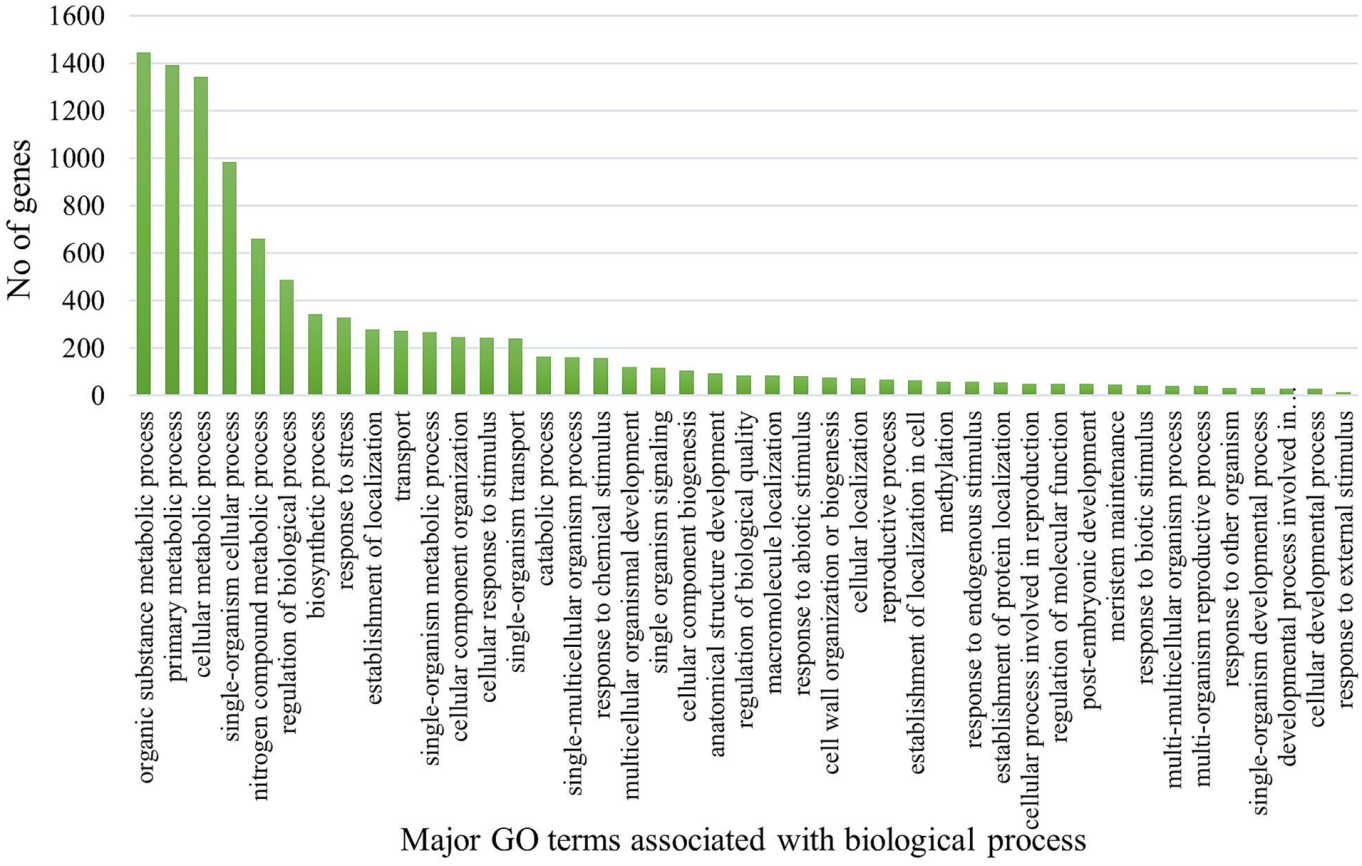
Distribution of genes across GO categories (biological process) in resistant genotype (EC0328964) upon infection of *B. sorokiniana*.

The gene ontology analysis was conducted to determine the functions of the potential differentially expressed genes (DEGs). In the resistant genotype (EC0328964) upon *B. sorokiniana* inoculation, 10,740 DEGs were attributed to biological process, 9,637 DEGs to the cellular component, and 7,591 DEG’s to molecular functions. A high proportion of DEGs were attributed to biological process in resistant genotype. In the biological process, most DEGs were attributed to the organic substance metabolic process (1,445), primary metabolic process (1,393), and cellular metabolic process (1,344), apart from that a high number of DEGs were attributed to response to stress and biotic stimulus (Figure 3). In the cellular component category, most of the DEGs were attributed to cell part (1,314), intracellular (1,125), intracellular part (1,122), and membrane part (1,048). In the molecular function category, most of the DEGs were attributed to organic cyclic compound binding (1,458), heterocyclic compound binding (1,458), ion binding (1,104), and transferase activity (675).

**Figure 4:**
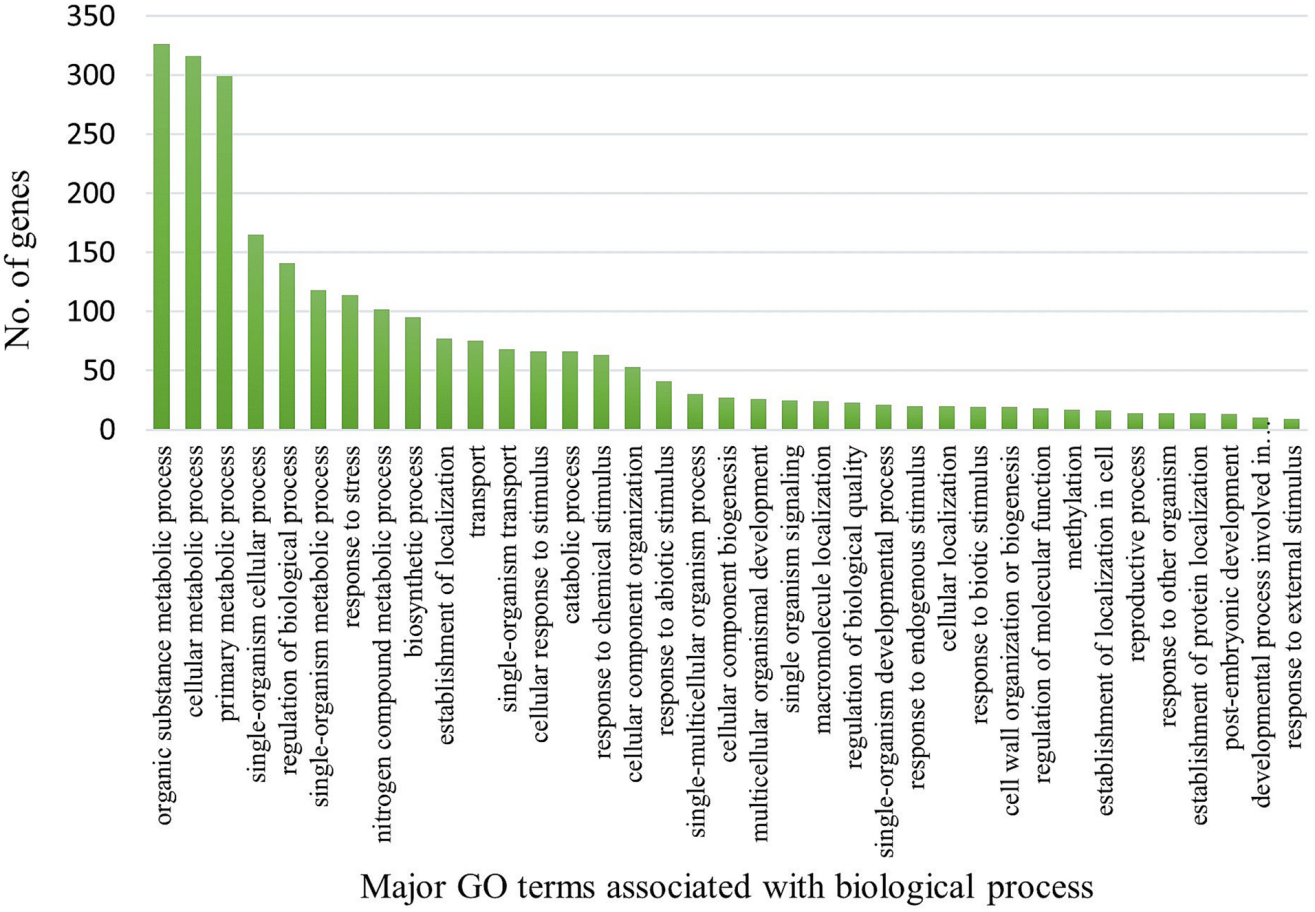
Distribution of genes across GO categories (biological process) in susceptible genotype (EC0578292) upon infection of *B. sorokiniana*.

**Figure 5:**
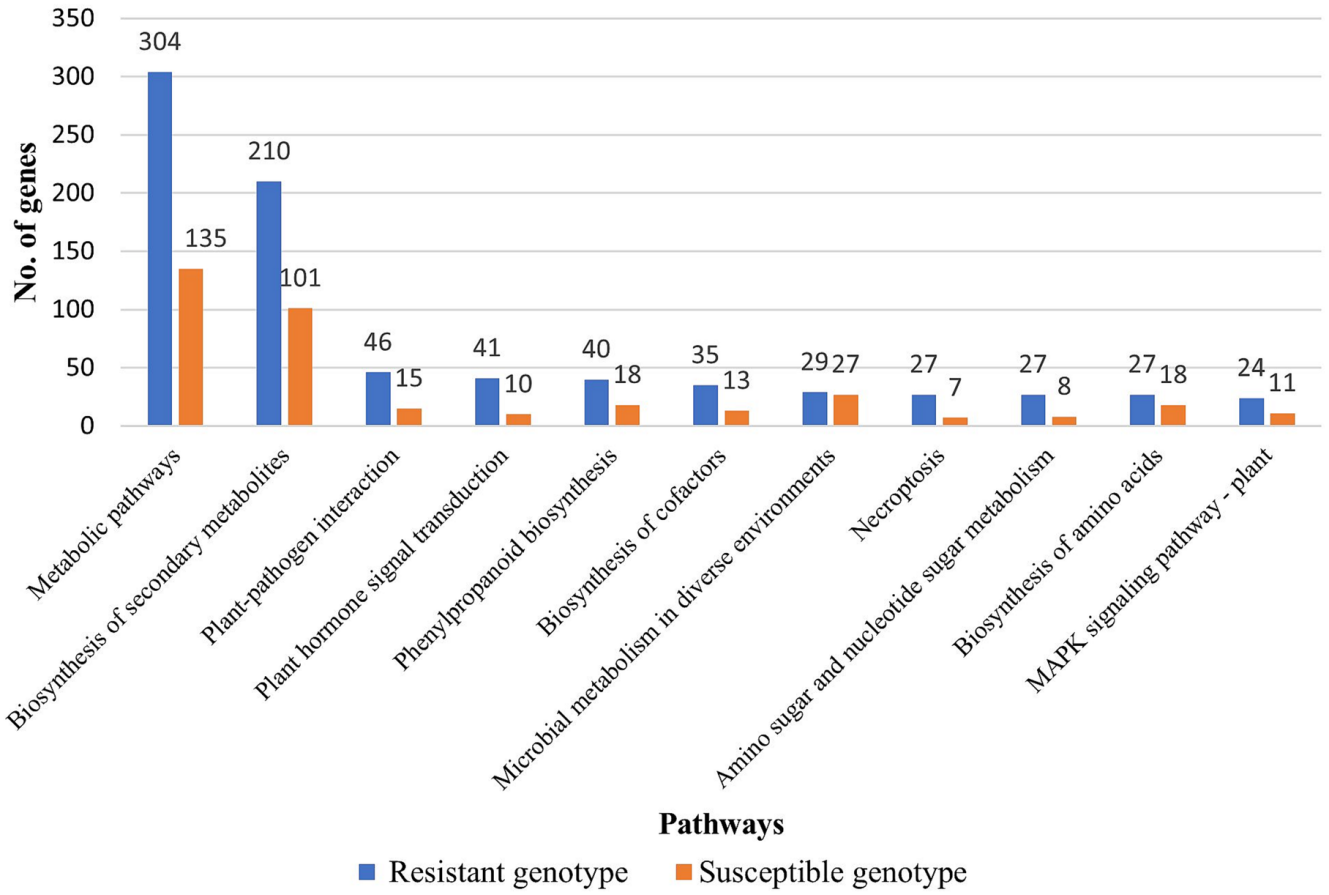
Major KEGG pathways identified in resistant and susceptible genotype of barley upon pathogen inoculation.

In the susceptible genotype (EC0578292), 2,618 DEGs were attributed to biological process, 2,704 DEGs were attributed to cellular component and 1,768 DEGs were attributed to molecular functions. The highest number of DEG’s were found in cellular components but almost an equal proportion to biological process in the susceptible genotype (Figure 4). In the biological process category, most DEGs were attributed to the organic substance metabolic process (326), cellular metabolic process (316), and primary metabolic process (299). In the cellular component category, most of the DEGs were attributed to cell part (392), intracellular (338), intracellular part (337), and intracellular organelle (270). In the molecular functions category, most of the DEGs were attributed to organic cyclic compound binding (280), heterocyclic compound binding (280), ion binding (272), and transferase activity (166). It indicated that a higher number of DEGs were present in the resistant genotype compared to the susceptible genotype pointing toward the role of genetic factors in governing resistance to spot blotch disease. In KEGG pathway analysis, metabolic pathways and biosynthesis of secondary metabolites were the most overrepresented in both the resistant and susceptible genotypes. However, the number of DEGs for both pathways (metabolic and biosynthesis of secondary metabolites) was higher in resistant than susceptible genotype (Supplementary Table S2). In addition, a higher number of DEGs in plant-pathogen interaction, plant hormone signal transduction, and MAPK signaling pathways were found in the resistant genotype (Figure 5).

#### 3.2.3 Defense-related genes in resistant genotype

The top up and down-regulated genes in the resistant genotype (EC0328964) were found based on log2 fold analysis. The highly up-regulated genes exhibited the functions *viz*. cation/H (+) antiporter-like, hypothetical protein, predicted protein, ribulose bisphosphate carboxylase, and hypothetical protein. The highly down-regulated genes function like Histone H3.2, low molecular mass early light-inducible protein HV90, ergosterol biosynthetic protein 28-like, histone H4, and high molecular mass early light-inducible protein HV58 (Table 2). The genes coding for defense responses in resistant genotype were identified based on GO terms like response to biotic stimulus, defense response, and response to stress in biological process (Table 3). Genes coding for RGAs (Resistance gene analogs) were highly upregulated in resistance genotypes like disease resistance protein RGA2-like (7-fold), disease resistance protein RGA5-like isoform X1 (6-fold), putative disease resistance protein RGA3 (6-fold), disease resistance protein RGA5-like isoform X1 (6-fold) and putative disease resistance protein RGA4 (6-fold). Other upregulated disease resistance protein were At1g50180 (7-fold), RPM1 (6-fold), RPH8A (6-fold), Pik-2-like (6-fold), PIK6-NP-like (6-fold), PIK6-NP (6-fold), NBS-LRR protein (4-fold), and *Pyricularia oryzae* resistance 21 protein (4-fold). Various PR proteins were also found to be upregulated like thaumatin-like protein PWIR2 (6-fold), pathogenesis-related protein 1(5-fold), pathogenesis-related protein PRB1-2-like (5-fold), and defensin Tm-AMP-D1.2-like (3-fold). Other genes like diacylglycerol kinase 4-like (5-fold), proline-rich protein HaeIII subfamily 1-like (5-fold), protein SRC2-like (4-fold), MLO protein homolog 1(4-fold) and transcription factor TCP14-like (3-fold) were found to be upregulated.

**Table 2:** Top differentially expressed genes based on log2 fold change in resistant genotype (EC0328964).

Top differentially expressed genes in resistant genotype	Log 2 fold change	Description/functions
Upregulated		
HORVU.MOREX.r3.6HG0634270.1	10.68	cation/H(+) antiporter 15-like
HORVU.MOREX.r3.4HG0373900.1	9.56	hypothetical protein ZWY2020_021451, partial
HORVU.MOREX.r3.6HG0571790.1	9.34	predicted protein
HORVU.MOREX.r3.4HG0357420.1	9.28	ribulose bisphosphate carboxylase /oxygenase activase B, chloroplastic
HORVU.MOREX.r3.7HG0719160.1	9.09	hypothetical protein ZWY2020_032988, partial
Downregulated		
HORVU.MOREX.r3.7HG0654460.1	−5.39	Histone H3.2
HORVU.MOREX.r3.5HG0482080.1	−5.38	low molecular mass early light-inducible protein HV90, chloroplastic-like
HORVU.MOREX.r3.6HG0592540.1	−5.08	ergosterol biosynthetic protein 28-like
HORVU.MOREX.r3.2HG0190220.1	−5.07	histone H4
HORVU.MOREX.r3.1HG0068490.1	−5.02	high molecular mass early light-inducible protein HV58, chloroplastic-like

**Table 3:** Significantly expressed defense-related genes exclusively in resistant (EC0328964) genotype.

Sl. No	Defense-related genes	Log 2 fold change	Descriptions	GO terms	KEGG Pathway
1	HORVU.MOREX.r3.1HG0084590.1	7.3	Disease resistance protein RGA2-like	P:defense response; F:ADP binding	
2	HORVU.MOREX.r3.7HG0749590.1	7.06	Putative disease resistance protein At1g50180	P:defense response; C:integral component of membrane; F:ADP binding	
3	HORVU.MOREX.r3.6HG0633870.1	6.47	Disease resistance protein RGA2-like	P:defense response; F:ADP binding	
4	HORVU.MOREX.r3.1HG0002960.1	6.44	Disease resistance protein RPM1	P:defense response; F:ADP binding	Plant-pathogen interaction;
5	HORVU.MOREX.r3.5HG0476860.1	6.23	Disease resistance protein RGA5-like isoform X1	P:defense response; F:ADP binding	-
6	HORVU.MOREX.r3.2HG0210300.1	6.21	Disease resistance protein Pik-2-like	P:defense response	-
7	HORVU.MOREX.r3.7HG0634540.1	6.18	Putative disease resistance protein RGA3	P:defense response; F:ADP binding	-
8	HORVU.MOREX.r3.3HG0311710.1	5.95	Disease resistance protein RPM1	P:defense response	-
9	HORVU.MOREX.r3.5HG0425330.1	5.95	Disease resistance protein RPH8A	P:defense response; F:ADP binding	-
10	HORVU.MOREX.r3.7HG0752010.1	5.88	Thaumatin-like protein PWIR2	P:defense response; P:metabolic process; P:response to biotic stimulus; F:glucan endo-1,3-beta-glucanase activity, C-3 substituted reducing group; F:glucan endo-1,4-beta-glucanase activity, C-3 substituted reducing group	-
11	HORVU.MOREX.r3.3HG0234700.1	5.86	Disease resistance protein RGA5-like isoform X1	P:defense response; F:ADP binding	-
12	HORVU.MOREX.r3.3HG0315570.1	5.73	Disease resistance protein PIK6-NP-like	P:defense response; F:ADP binding	-
13	HORVU.MOREX.r3.5HG0501830.1	5.72	Disease resistance protein PIK6-NP	P:defense response; F:ADP binding	-
14	HORVU.MOREX.r3.7HG0747390.1	5.6	Putative disease resistance protein RGA4	P:defense response; F:ADP binding	-
15	HORVU.MOREX.r3.7HG0696660.1	5.36	Diacylglycerol kinase 4-like	F:NAD+ kinase activity; F:diacylglycerol kinase activity; F:ATP binding; P:defense response; P:protein kinase C-activating G protein-coupled receptor signaling pathway; P:phosphorylation	Metabolic pathways; Biosynthesis of secondary metabolites; Glycerolipid metabolism; Glycerophospholipid metabolism; Axon regeneration; Choline metabolism in cancer;
16	HORVU.MOREX.r3.7HG0634640.1	5.29	Putative disease resistance protein RGA3	P:defense response; F:ADP binding	
17	HORVU.MOREX.r3.7HG0668880.1	5.06	Pathogenesis-related protein PRB1-2-like	C:extracellular space; P:defense response; P:response to biotic stimulus	MAPK signaling pathway - plant; Plant hormone signal transduction; Plant-pathogen interaction;
18	HORVU.MOREX.r3.5HG0473560.1	4.84	Pathogenesis-related protein 1	C:extracellular space; P:defense response; P:response to biotic stimulus	MAPK signaling pathway - plant; Plant hormone signal transduction; Plant-pathogen interaction;
19	HORVU.MOREX.r3.7HG0748550.1	4.75	Proline-rich protein HaeIII subfamily 1-like	P:defense response	-
20	HORVU.MOREX.r3.5HG0473580.1	4.73	Pathogenesis-related protein 1	C:extracellular space; P:defense response; P:response to biotic stimulus	MAPK signaling pathway - plant; Plant hormone signal transduction; Plant-pathogen interaction;
21	HORVU.MOREX.r3.2HG0113980.1	4.35	protein SRC2-like	P:defense response	-
22	HORVU.MOREX.r3.4HG0410660.1	4.3	MLO protein homolog 1	F:calmodulin binding; P:defense response; P:response to biotic stimulus; C:integral component of membrane	-
23	HORVU.MOREX.r3.2HG0095540.1	3.98	Protein *Pyricularia Oryzae* resistance 21	F:metal ion binding; P:regulation of defense response to fungus	
24	HORVU.MOREX.r3.2HG0198100.1	3.52	NBS-LRR disease resistance protein	P:defense response; F:ADP binding	-
25	HORVU.MOREX.r3.6HG0615130.1	3.49	Transcription factor TCP14-like	F:transcription cis-regulatory region binding; F:DNA-binding transcription factor activity; C:nucleus; P:regulation of DNA-templated transcription; P:regulation of defense response	-
26	HORVU.MOREX.r3.1HG0002530.1	3.16	Defensin Tm-AMP-D1.2-like	P:killing of cells of another organism; P:defense response to fungus	-

### 3.3 Analysis of virulence genes of *Bipolaris sorokiniana*

#### 3.3.1 Differential gene expression analysis, gene ontology and functional annotation

**Figure 6:**
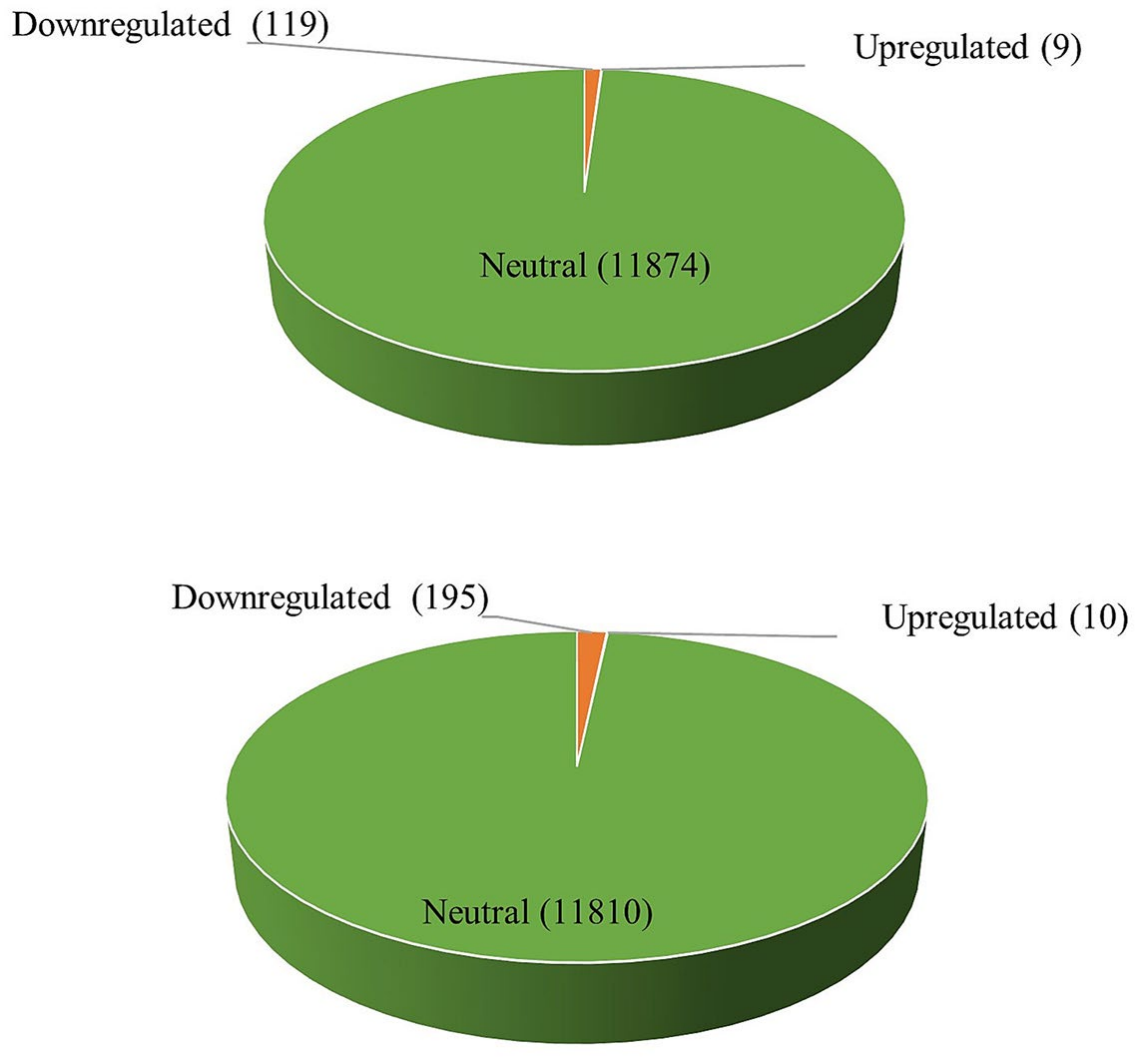
Differential gene expression analysis of *Bipolaris sorokiniana* inoculated in (top) resistant host genotype background (EC0328964) and (bottom) susceptible host genotype background (EC0578292).

**Figure 7:**
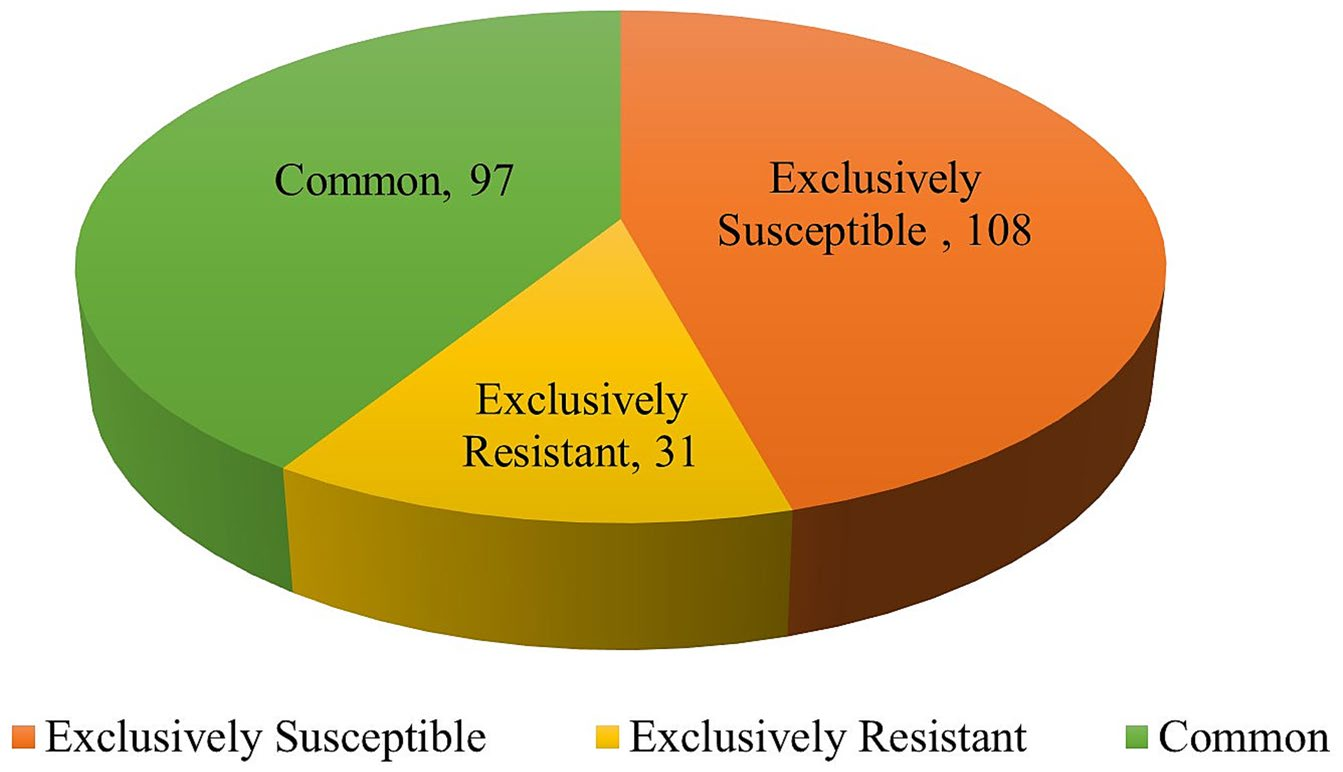
Common and unique genes of *Bipolaris sorokiniana* identified upon inoculation in two different host genotype backgrounds.

The differentially expressed genes of *B*. *sorokiniana* were analyzed in both the combinations of *B*. *sorokiniana* grown *in vitro*-resistant inoculated (BS_RI) and *B*. *sorokiniana* grown *in vitro*-susceptible inoculated (BS_SI). In the resistant genotype (EC0328964) background, 128 genes were differentially expressed. Out of the 128 genes, 9 genes were upregulated, and 119 genes were downregulated (Figure 6). In the susceptible genotype (EC0578292) background, 205 genes were differentially expressed with a *p*-value of ≤0.05. Out of 205 genes, 10 genes were upregulated, and 195 genes were downregulated. In the two combinations, 97 genes were commonly expressed, 31 genes were exclusively expressed in resistant genotype and 108 genes were exclusively expressed in susceptible genotype (Figure 7) (Supplementary Table S3).

**Figure 8:**
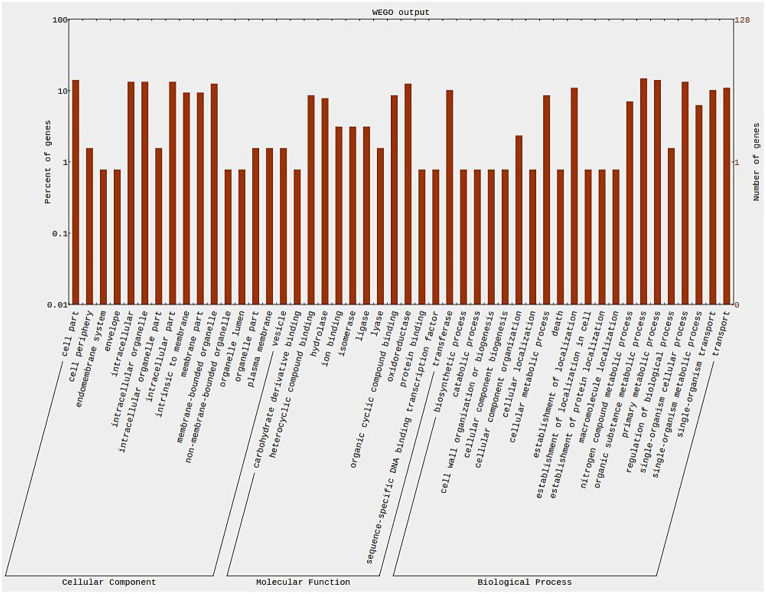
Wego plot showing distribution of *Bipolaris sorokiniana* genes in resistant host genotype background across three categories.

**Figure 9:**
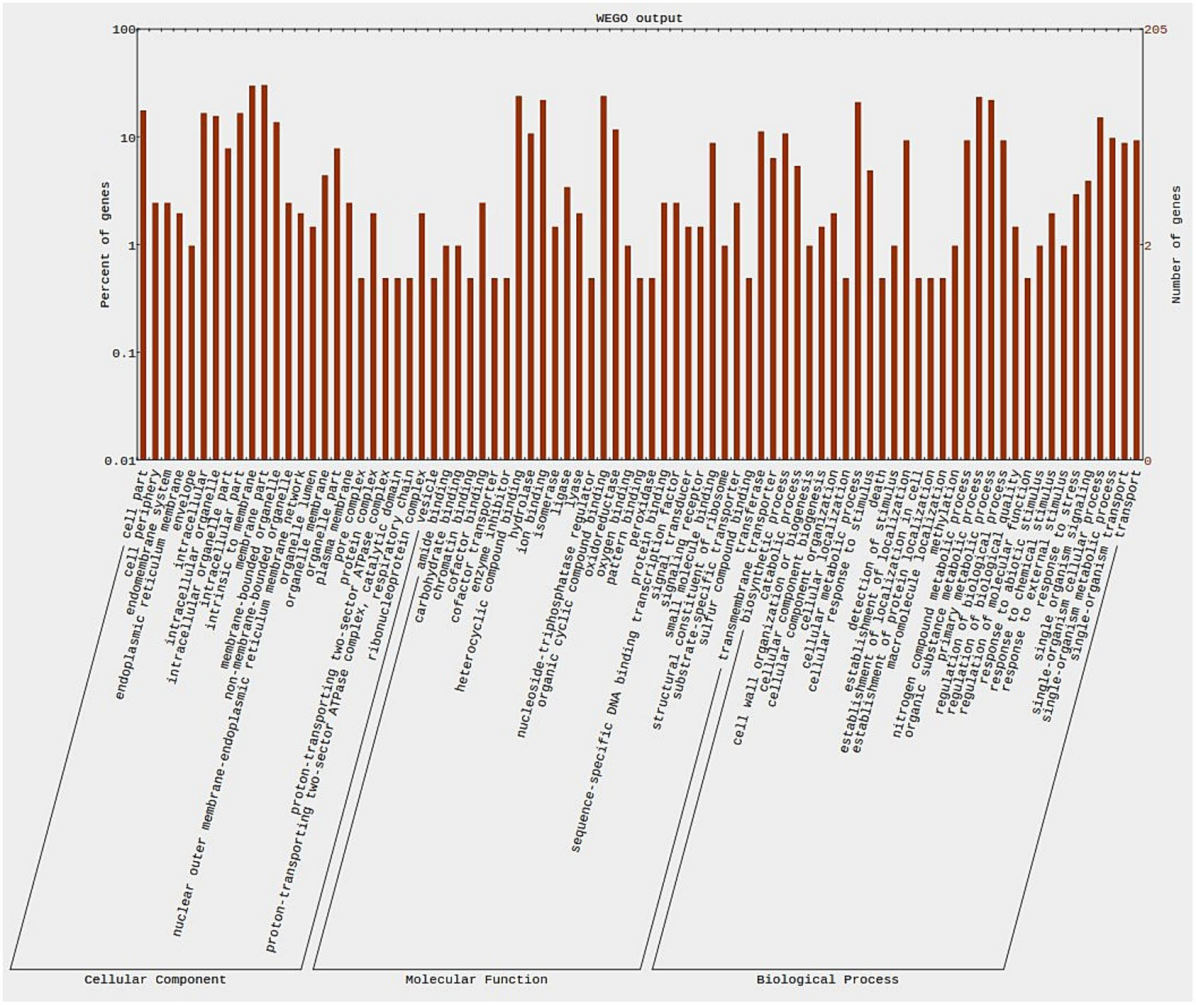
Wego plot showing distribution of *Bipolaris sorokiniana* genes in Susceptible host genotype background across three categories.

**Figure 10:**
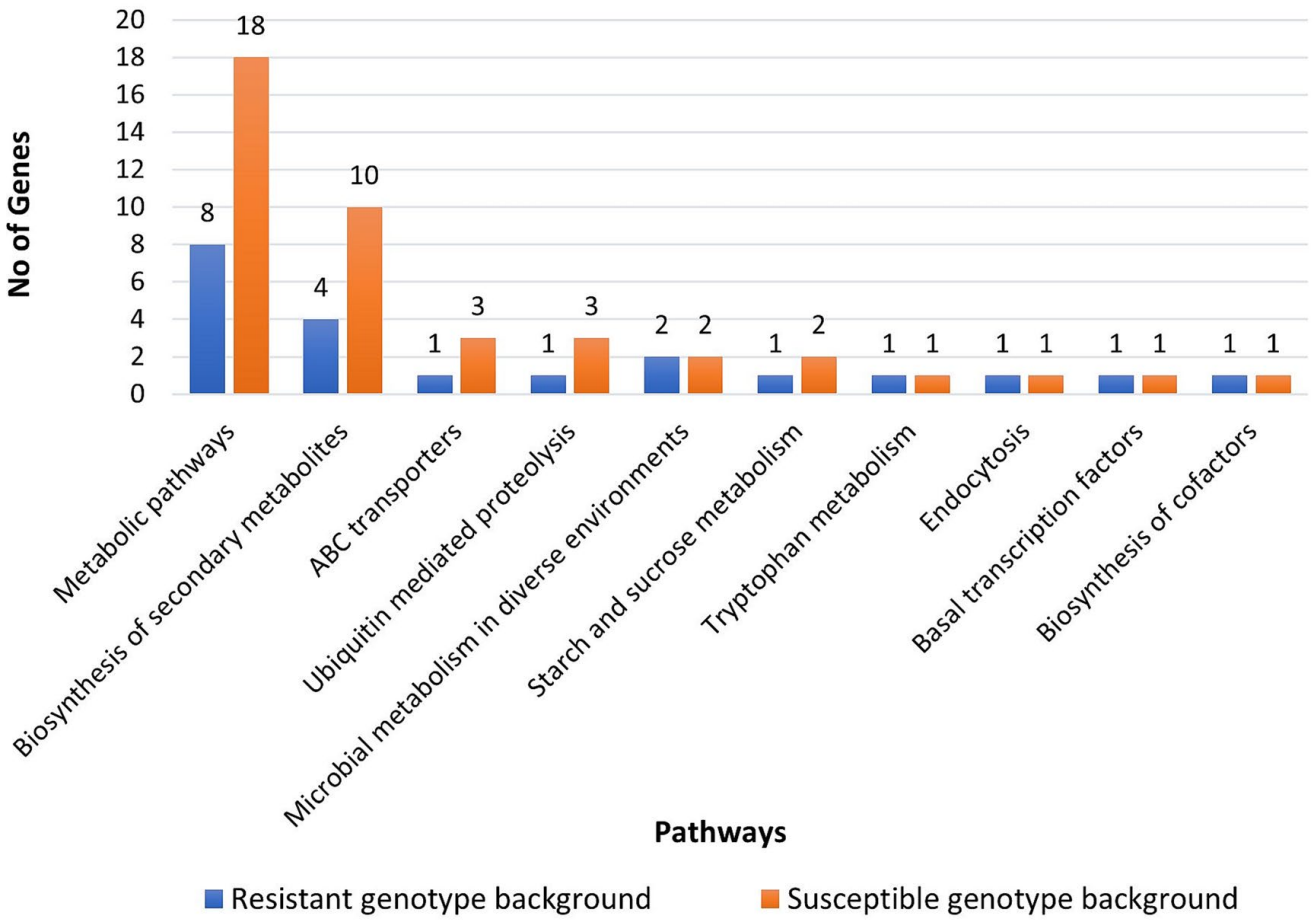
Major KEGG pathways identified in *Bipolaris sorokiniana* upon inoculation in resistant and susceptible host genotype.

In the resistant genotype (EC0328964) background, 137 DEGs were attributed to biological process, 123 DEGs to cellular component and 78 DEGs to molecular functions. In the biological process, most DEGs were attributed to the organic substance metabolic process (19), primary metabolic process (18), and single organism cellular process (17). In the cellular component category, most of the DEGs were attributed to cell part (18), intracellular (17), intracellular part (17), and intracellular organelles (17). In the molecular function category, most of the DEGs were attributed to oxidoreductase activity (16), transferase activity (13), heterocyclic compound binding (11), and organic cyclic compound binding (11) (Figure 8). In the susceptible genotype (EC0578292) background, 374 DEGs were attributed to cellular component, 368 DEGs were attributed to biological process and 298 DEGs were attributed to molecular functions. In the cellular component category, most of the DEGs were attributed to the membrane part (62), intrinsic to membrane (61), cell part (36), and intracellular (34). In the biological process category, most DEGs were attributed to the organic substance metabolic process (48), primary metabolic process (45), and cellular metabolic process (43). In the molecular functions category, most of the DEGs were attributed to organic cyclic compound binding (49), heterocyclic compound binding (49), ion binding (45), and oxidoreductase activity (24) (Figure 9) (Supplementary Table S4). Higher number of putative virulence genes were differentially expressed in the susceptible genotype. Upon KEGG pathway analysis, numerous pathways were enriched in the susceptible host. Metabolic pathways and biosynthesis of secondary metabolites were the most overrepresented pathways in both the resistant and susceptible host backgrounds (Figure 10). The genes encoding for ABC transporters and Ubiquitin mediated proteolysis pathways were found to be more in susceptible host indicating its probable role in the infection process.

#### 3.3.2 Genes with probable role in pathogenesis and possible candidate effectors

A higher number of genes were found downregulated than upregulated in both resistant and susceptible genotype backgrounds. The top 10 differentially expressed genes were analyzed for both the host backgrounds (Table 4). The gene encoding for HET domain-containing protein was highly upregulated (8.8–8.5-fold) irrespective of the host genetic background suggesting its involvement in pathogen functionality. CENPB Domain containing protein, which is involved in DNA binding activity was found specifically upregulated (8.4-fold) in susceptible host background. The P-type cation-transporting protein-encoding gene having hydrolase activity was specifically upregulated (8.2-fold) in resistant host background. The specificity of genes being regulated implies altered pathogen functions when inoculated in two contrasting host genetic backgrounds. Pyruvate decarboxylase and hexose transporter HXT13 were among the highly downregulated genes in both the host genetic backgrounds.

**Table 4:** Top up and down-regulated genes of *B*. *sorokiniana* upon inoculation in two different host genotype backgrounds.

Genes	log2 fold change	Description
Susceptible genotype background		
EMD59272	8.53	HET-domain-containing protein
EMD62759	8.42	CENPB domain containing protein
EMD68918	−7.94	pyruvate decarboxylase
EMD63308	−7.15	zinc finger transcription factor
EMD60302	−6.96	Hexose transporter HXT13
EMD66098	−6.50	HSP20-like chaperone
EMD65754	−6.41	Zinc knuckle
EMD67510	−6.37	glycosyltransferase family 4 protein
EMD69284	−6.32	FAS1 domain-containing protein
EMD58070	−6.27	retropepsin-like domain-containing protein
Resistant genotype background		
EMD59272	8.80	HET-domain-containing protein
EMD61245	8.20	P-type cation-transporting
EMD60302	−7.79	Hexose transporter HXT13
EMD68918	−7.32	pyruvate decarboxylase
EMD61266	−7.13	NRDE protein-domain-containing protein
EMD62119	−6.67	l-serine dehydratase
EMD61303	−6.46	uncharacterized protein COCSADRAFT_39039
EMD58945	−6.36	siderophore iron transporter mirC
EMD63022	−6.34	cdp-alcohol phosphatidyltransferase
EMD69143	−6.31	uncharacterized protein COCSADRAFT_31904

**Figure 11:**
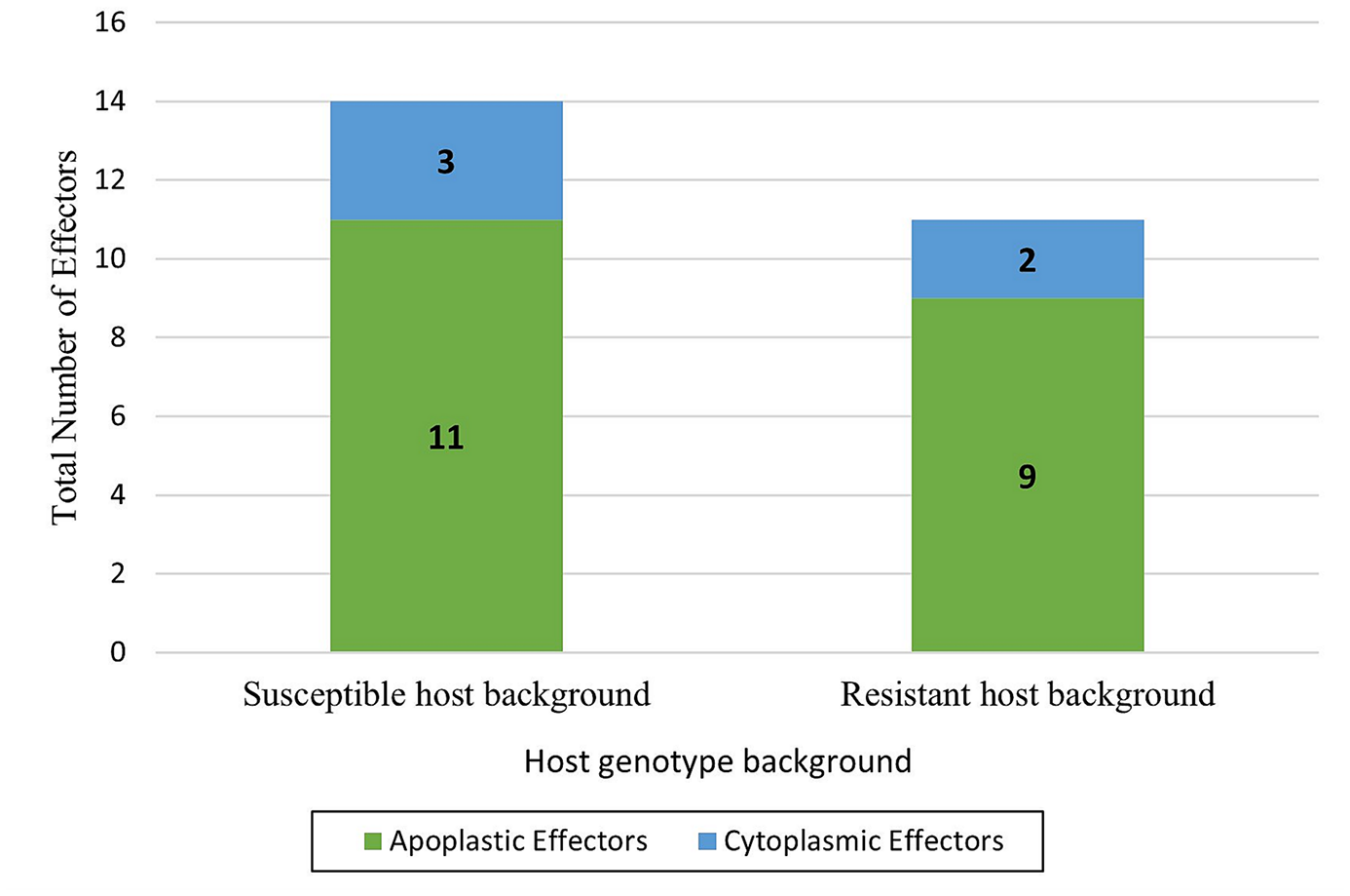
Candidate effectors (Apoplastic and Cytoplasmic) identified in *Bipolaris sorokiniana* upon inoculation in two different host genotype backgrounds.

205 differentially expressed genes of *B*. *sorokiniana* in the susceptible background were analyzed for the presence of N-terminal signal peptide. 42 gene sequences were predicted as signal peptides. 41 sequences were predicted as signal peptides with no subcellular localization. To check for the presence of transmembrane domain, 41 sequences were subjected to TMHMM 2.0 out of which 33 were predicted as non-transmembrane secreted proteins. The 33 sequences were then further analyzed using the Effector P 3.0 which predicted 14 gene sequences (11 apoplastic and 3 cytoplasmic) as effectors (Table 5). The identified sequences belonged to families *viz*. hydrolases, GPI anchored serine–threonine rich protein, FAS1 domain-containing protein, etc. Similarly, the presence of signal peptides was analyzed from 128 differentially expressed genes of *B*. *sorokiniana* in the resistant host. Sequentially, 29, 28, 22 and 11 sequences were identified by Signal P 5.0, Target P 2.0, TMHMM 2.0, and EffectorP 3.0, respectively. Out of which, 11 sequences were identified as effectors, 2 were denoted as cytoplasmic effectors and 9 were apoplastic effectors (Figure 11) (Supplementary Table S5). All the identified effectors except one (nad mitochondrial precursor) were also identified in the susceptible host.

**Table 5:** The candidate effector proteins.

Gene Id	Description	Effector
Susceptible host background		
EMD69701	glycoside hydrolase family 16 protein	Apoplastic effector
EMD69284	FAS1 domain-containing protein	Cytoplasmic effector
EMD69264	NAC-domain-containing protein	Apoplastic effector
EMD66829	c4-dicarboxylate transporter malic acid transport protein	Apoplastic effector
EMD66725	atp-dependent rna helicase dbp9	Apoplastic effector
EMD64635	antigenic thaumatin-like protein	Apoplastic/ cytoplasmic effector
EMD64479	uncharacterized protein COCSADRAFT_37061	Cytoplasmic/Apoplastic effector
EMD63848	uncharacterized protein COCSADRAFT_37598	Apoplastic effector
EMD63454	uncharacterized protein COCSADRAFT_37239	Apoplastic effector
EMD63612	Asp f 13-like protein	Apoplastic effector
EMD62743	victoriocin	Apoplastic effector/ Cytoplasmic effector
EMD60725	putative ricin b lectin protein	Cytoplasmic effector
EMD60043	gpi anchored serine–threonine rich protein	Apoplastic effector
EMD59947	Acetyl-CoA synthetase-like protein	Apoplastic effector
Resistant host background		
EMD69701	glycoside hydrolase family 16 protein	Apoplastic effector
EMD69284	FAS1 domain-containing protein	Cytoplasmic effector
EMD69264	NAC-domain-containing protein	Apoplastic effector
EMD66829	c4-dicarboxylate transporter malic acid transport protein	Apoplastic effector
EMD66754	nad mitochondrial precursor	Apoplastic effector
EMD63848	uncharacterized protein COCSADRAFT_37598	Apoplastic effector
EMD63454	uncharacterized protein COCSADRAFT_37239	Apoplastic effector
EMD63612	Asp f 13-like protein	Apoplastic effector
EMD62743	victoriocin	Apoplastic effector/ Cytoplasmic effector
EMD60725	putative ricin b lectin protein	Cytoplasmic effector
EMD60043	gpi anchored serine–threonine rich protein	Apoplastic effector

## 4 Discussion

Plant–pathogen interaction is an intricate process that initiates numerous molecular reactions at various stages of infection (Peyraud et al., 2017). Analysis of transcriptome is a useful technique to decipher complex underlying processes governing plant-pathogen interaction. *B. sorokiniana* is a devastating pathogen causing spot blotch of wheat and barley. Untill now, no information is available on the interaction between *B. sorokiniana* and *H. vulgare*. Keeping this in view, an RNA sequencing approach was employed to analyse the transcriptome of the barley-*B. sorokiniana* interaction in both the resistant (EC0328964) and susceptible (EC0578292) genotypes. This investigation was carried out to gain insights into the resistance mechanisms in the host and the pathogen factors contributing to virulence.

Plants defend themselves by activating the defense response, which is triggered by the recognition of molecules of pathogen origin (De Wit et al., 2009). In the present study, several disease-resistant proteins of *Hordeum vulgare* were identified in the resistant genotype (EC0328964). The genes encoding for RGAs (resistant gene analogs) like disease resistance protein RGA2-like (7-fold), disease resistance protein RGA5-like isoform X1 (6-fold), putative disease resistance protein RGA3 (6-fold), and putative disease resistance protein RGA4 (6-fold) were found to be highly upregulated and present exclusively in resistant genotype in response to *B*. *sorokiniana*. Earlier studies revealed that these RGAs shared sequence similarities to the known R genes and were present abundantly in the genome of many plants (McDowell and Simon, 2006). Later, RGAs were involved in the resistance mechanisms against different pathogens (Zhang et al., 2013; Islam et al., 2020). The present investigation indicated an important role of RGAs in governing resistance in barley against *B*. *sorokiniana*. Pathogenesis-related proteins, which are involved in defense responses in the host, were also highly upregulated in the resistance genotype. The genes like pathogenesis-related protein PRB1-2-like (5-fold) pathogenesis-related protein 1(5-fold), thaumatin-like protein PWIR2 (6-fold), and defensin Tm-AMP-D1.2-like protein (3-fold) were highly expressed in resistant genotype only. Other PR proteins like chitinase 8 like were found in both the resistant and susceptible genotypes but were highly expressed in the resistant as compared to the susceptible genotype. In a recent study, through transcriptome analysis, the PR proteins like PR1, Chitinase 11, and defensin were reported slightly upregulated in the susceptible genotype than highly susceptible genotype of wheat in response to *B. sorokiniana* pathogen (Li et al., 2021). Thaumatin-like proteins (PR-5 family) have been earlier reported to be upregulated during plant-pathogen interaction (Singh et al., 2013). RPM1 is a peripheral membrane protein, which governs resistance to the pathogen *Pseudomonas syringae* (Boyes et al., 1998). In the present study, RPM1 protein (6-fold) was found to be upregulated in resistant genotype. The gene governing resistance to powdery mildew *mlo* (recessive) renders the plant susceptible to *B. sorokiniana* and other pathogens like *Ramularia collo-cygni*, *Magnaporthe grisea*, and *Fusarium graminearum* (Jarosch et al., 1999; Kumar et al., 2001; Jansen et al., 2005; McGrann et al., 2014). Thus, the presence of *MLO* (dominant/wild type) gene contributes to the defense responses against *B*. *sorokiniana*. MLO protein (4-fold) was upregulated and exclusively present in resistant genotypes in our study. Similarly, RNA-Seq analysis of barley- *Ramularia collo-cygni* interaction, Lemcke et al. (2021) revealed that MLO proteins have a role in resistance. Diacylglycerol kinase (DGK) is a pivotal enzyme within plant lipid signaling, holds significance in a plant’s metabolic framework, and influences its reaction to diverse external stresses (Carther et al., 2020). DGK proteins have been increased upon pathogen infection in crops like tomato and rice (Young et al., 1996; Van Der Luit et al., 2000). In our study, diacyl-glycerol kinase protein (5-fold) was upregulated in the resistant genotype. The disease-resistant PIK protein is known to play a role in interaction with the effector protein in the rice-*Magnaporthe oryzae* pathosystem (Zhai et al., 2011). In this study, genes similar to disease resistance PIK protein (6-fold) were expressed in resistant host. Based on GWAS in barley-*B*. *sorokiniana* reported that most QTLs mapped to genes coding NBS-LRR proteins (Visioni et al., 2020). Yazawa et al. (2013) conducted transcriptome analysis in *B. sorghicola* -sorghum pathosystem and found LRR family receptors to be involved in defense mechanisms. We also found NBS-LRR protein (4-fold) upregulated in the resistant genotype. TCP transcription factors serve as a cellular hub in plant defense signaling and stimulate the biosynthetic pathways like brassinosteroid (BR), jasmonic acid (JA), and flavonoids (Kim et al., 2014; Li, 2015). TCP-14-like transcription factor (4-fold) was found to be upregulated in resistant genotype in our study. Overall, the genetic basis of resistance of *H. vulgare* toward spot blotch might be due to a variety of genes having defense-related activities.

In the present investigation, gene ontology analysis conducted revealed several GO term representations in both the susceptible and resistant genotypes. In the biological process category, most of the DEGs were found to be involved in the metabolic process. Previous studies reported that the transcriptome of wheat-*Tilletia indica* pathosystem had a high number of DEGs attributed to metabolic process (Gurjar et al., 2022). In the molecular function and cellular component category, most of the DEGs were involved in binding and cell part, respectively. Similar results have been obtained in earlier reports (Gurjar et al., 2022). In our study, KEGG pathway analysis revealed predominantly metabolic pathways, with biosynthesis of secondary metabolites present in both the resistant and susceptible genotypes. Earlier, transcriptomes of *Arabidopsis* in response to different pathogens (*Pseudomonas syringae* pv. *maculicola*, *Hyaloperonospora arabidopsidis*, *Fusarium oxysporum*, *Pseudomonas syringae*, Cabbage leaf curl virus, *Botrytis cinerea*, *Pseudomonas syringae*) revealed metabolic pathways and biosynthesis of secondary metabolites as the most overrepresented pathways (Biniaz et al., 2022). The role of metabolic plant responses and secondary metabolites during plant-pathogen interaction have been reported (Bednarek, 2012). In our study, the MAPK signaling pathway, plant-pathogen interaction, and plant hormone signal transduction were more enriched in resistant (EC0328964) than susceptible genotype (EC0578292) during barley-*B*. *sorokiniana* interaction. Earlier studies on other host-pathogen in barley-*Blumeria graminis* pathosystem have shown that mitogen-activated protein kinase (MAPK) signaling and plant hormone signaling pathways are upregulated in resistant genotype (Li et al., 2019). In our opinion, the DEGs associated with the MAPK signaling pathway were more in the resistant genotype than the susceptible genotype indicating an important role of this pathway in spot blotch resistance mechanism in barley. Further, MAPK signaling pathway genes will be characterized using the functional genomics approach.

Plant pathogens have several strategies for the production of cell wall degrading enzymes, secondary metabolites, and secreted proteins to counteract plant defense responses (De Wit et al., 2009; Kubicek et al., 2014). The intricate mechanisms underlying the interaction between *B*. *sorokiniana* and *Hordeum vulgare* is poorly understood and it is confined to *Triticum aestivum-B. sorokiniana* pathosystem. In this study, we identified a few genes involved in the pathogenicity of *B*. *sorokiniana*. In both the host genetic backgrounds, most of the pathogen genes were downregulated rather than upregulated. The functional annotation revealed that in the susceptible host genotype background the pathways governing metabolism, biosynthesis of secondary metabolites, ABC transporters, and Ubiquitin mediated proteolysis were highly enriched in comparison to the resistant host genotype background. This indicates that the susceptible host promotes the activities of the pathogen and provides a suitable environment for successful colonization. In earlier reports, secondary metabolites aid the pathogens to successfully evade plant defense responses (Keller et al., 2005). ABC transporters too play an important role in safeguarding pathogens against plant defense compounds (Yazaki, 2006). In the present study, upregulation of gene encoding for HET domain-containing was observed in both the genotypes. The HET domain contains proteins highly abundant in fungal genomes and attributed to governing incompatibility systems (Paoletti and Clave, 2007). With emerging studies, these proteins might have other functions as well and likely play a role in fungal immunity (Daskalov, 2023). However, most of the HET domain proteins remain unannotated and their probable roles during *in-planta* interactions are yet to be deciphered. In the present study, the gene encoding CENPB domain-containing protein was noticed to be exclusively upregulated in susceptible genotype background. In earlier reports, CENPB proteins were responsible for centromere formation in mammals (Masumoto et al., 1989; Fachinetti et al., 2015). Homologs for CENPB protein have been reported in *Neurospora crassa* but its function remains unknown (Smith et al., 2012). Enzymes responsible for hydrolase, oxidoreductase, and transferase activity were found to be downregulated in our study. These enzymes play an important role in various reaction cycles. Glycoside hydrolase represents the largest family of hydrolase enzymes, which is responsible for enzymatic activity in degrading plant cell walls. In earlier reports, glycoside hydrolase proteins were downregulated in the biotrophic phase of *Cladosporium fulvum* and upregulated during necrotrophic phase (Okmen et al., 2019). Effectors are traditionally classified as small cysteine-rich, transmembrane domains lacking proteins along with signal peptide domains (Zhang et al., 2020). In the present study, 14 sequences were identified as candidate effectors in the susceptible host and 11 effectors in the resistant host background, which belonged to families like hydrolase, fascilin domain protein, and other uncharacterized proteins. Earlier, Fasciclin/FAS1 family proteins have been reported as cell adhesion molecules in fungi and other organisms (Miyazaki et al., 2007). Liu et al., 2009 identified a fasciclin-like protein (MoFLP1) involved in conidiation, conidia adhesion, and pathogenicity in *Magnaporthe oryzae*. The glycoside hydrolase family 16 protein (GH16) was also identified as apoplastic effectors in both the barley genotypes. Plant pathogens deploy an arsenal of effectors which are expressed in a spatio-temporal manner, thereby making it essential to identify its expression across various tissues and time points (Toruno et al., 2016).

## 5 Conclusion

Transcriptomic analysis of barley genotypes with challenge inoculation of *B*. *sorokiniana* was performed using the RNA Seq approach and defense-related genes and pathogenicity determinants involved during *Hordeum vulgare*-*B*. *sorokiniana* interaction were identified. The defense-related genes *viz*., RGA2-like (7-fold), disease resistance protein RGA5-like isoform X1 (6-fold), putative disease resistance protein RGA3 (6-fold) and putative disease resistance protein RGA4 (6-fold) were highly expressed in resistant genotype only. 14 effectors of B. *sorokiniana* were identified *viz*. 3 cytoplasmic and 11 apoplastic effectors in resistant host background. The pathways encoding for metabolism, biosynthesis of secondary metabolites, ABC transporters, and Ubiquitin mediated proteolysis were higher in susceptible genotype. This is the first report elucidating the transcriptome of barley-*B*. *sorokiniana* interaction which might provide valuable information on genetic factors involved in the spot blotch resistance of barley.

## Data availability statement

The datasets presented in this study are deposited in the NCBI database under accession number PRJNA996376 (SRR25379257, SRR25379256, SRR25379255, SRR25379254, SRR25379253, SRR25379252, SRR25379251, SRR25379250, SRR25379249, SRR25379248).

## Author contributions

PB: Data curation, Formal analysis, Investigation, Methodology, Software, Writing – original draft. MG: Conceptualization, Funding acquisition, Supervision, Writing – original draft, Writing – review & editing. TK: Data curation, Methodology, Writing – review & editing. NK: Data curation, Formal analysis, Writing – original draft. DS: Data curation, Methodology, Writing – review & editing. SJ: Formal analysis, Methodology, Writing – review & editing. MS: Methodology, Project administration, Writing – review & editing.

## Footnotes

^1^ https://www.blast2go.com

^2^ https://www.genome.jp/kegg/kaas/

^3^ https://services.healthtech.dtu.dk/services/SignalP-5.0/

^4^ https://services.healthtech.dtu.dk/services/TargetP-2.0/

^5^ https://services.healthtech.dtu.dk/services/TMHMM-2.0/

^6^ https://effectorp.csiro.au/
